# Putative novel mediators of acute kidney injury in critically ill patients: handling by continuous venovenous hemofiltration and effect of anticoagulation modalities

**DOI:** 10.1186/s12882-015-0167-5

**Published:** 2015-10-30

**Authors:** Louise Schilder, S. Azam Nurmohamed, Pieter M. ter Wee, Nanne J. Paauw, Armand RJ Girbes, Albertus Beishuizen, Robert HJ Beelen, AB Johan Groeneveld

**Affiliations:** Department of Nephrology, VU University medical center, De Boelelaan 1117, 1081 HV Amsterdam, The Netherlands; Department of Molecular Cell Biology and Immunology, Netherlands, The Netherlands; Department of Intensive Care, VU University Medical Center, Amsterdam, The Netherlands; Department of Intensive Care, Medisch Spectrum Twente, Enschede, The Netherlands; Department of Intensive Care, Erasmus Medical Center, Rotterdam, The Netherlands

**Keywords:** Acute kidney injury, Citrate, Continuous venovenous hemofiltration, Critically ill patients, TWEAK, Angiopioetin-2, Pentraxin-3

## Abstract

**Background:**

Novel putative mediators of acute kidney injury (AKI) include immune-cell derived tumour necrosis factor-like weak inducer of apoptosis (TWEAK), angiopoietin-2 (Ang-2) and protein pentraxin-3 (PTX3). The effect of continuous venovenous hemofiltration (CVVH) and different anticoagulation regimens on plasma levels were studied.

**Methods:**

At 0, 10, 60, 180 and 720 min of CVVH, samples were collected from pre- and postfilter blood and ultrafiltrate. No anticoagulation (*n* = 13), unfractionated heparin (*n* = 8) or trisodium citrate (*n* = 21) were compared.

**Results:**

Concentrations of TWEAK, Ang-2 and PTX3 were hardly affected by CVVH since the mediators were not (TWEAK, PTX3) or hardly (Ang-2) detectable in ultrafiltrate, indicating negligible clearance by the filter in spite of molecular sizes (TWEAK, PTX3) at or below the cutoff of the membrane. Heparin use, however, was associated with an increase in in- and outlet plasma TWEAK.

**Conclusion:**

Novel AKI mediators are not cleared nor produced by CVVH. However, heparin anticoagulation increased TWEAK levels in patient’s plasma whereas citrate did not, favouring the latter as anticoagulant in CVVH for AKI.

## Background

Acute kidney injury (AKI) is common in critically ill patients, particularly in case of sepsis, and is associated with increased morbidity and mortality even in the long term [1]. Although the pathogenesis of AKI is multifactorial, novel mediators have been described that are implicated in the development of AKI, for instance in the course of sepsis. These mediators include TNF-associated weak inducer of apoptosis (TWEAK), a member of the super tumor necrosis factor (TNF) family, which can be expressed in renal and immune cells [2, 3]. Also, angiopoietin-2 (Ang-2), a proinflammatory and endothelial barrier-destabilizing mediator in the vessel wall and macrophages, has been described to play a role in AKI and has been reported to be a predictor of mortality in patients with dialysis-dependent AKI [4–6]. Finally, pentraxin-3 (PTX3) is an acute phase protein located in endothelium and stored in neutrophils, that recently is considered to play a role in the pathogenesis of ischemic acute kidney injury, although renal protective effects have been described as well [7, 8].

Continuous venovenous hemofiltration (CVVH) is often applied in the treatment of AKI and clearance and absorption by the filter membrane of harmful substances, dependent on molecular weight and membrane properties among others, remain a controversial issue. It has been postulated that citrate, used for filter anticoagulation, prevents, partly by chelating calcium, degranulation of neutrophils induced by systemic heparin following complement activation and blood filter contact during CVVH [9, 10]. Hence, the anticoagulation regimen applied during CVVH could influence release of proinflammatory factors and specific AKI mediators by activated immune cells and platelets in the patient and hemofilter.

The aim of the current study was to assess how the novel, putative mediators of AKI are handled by CVVH and whether different anticoagulation regimens have additional effects, in critically ill patients with AKI.

## Methods

The patient population and methods for this study are reported elsewhere [10, 11]. Study protocols were approved by the local medical ethical committee and performed in accordance with the Declaration of Helsinki. Patients were recruited during office hours for sampling when participating in one of the two following studies. The first study started 1 year before the availability of a custom-made citrate-based replacement fluid. From March 2004 to September 2005, patients admitted to the intensive care unit (ICU) who developed AKI necessitating CVVH but in whom heparin was contraindicated due to a high bleeding tendency, were either treated by anticoagulant-free CVVH (*n* = 13) or, after becoming available in 2005, by regional citrate anticoagulation (*n* = 10) and were prospectively followed [12]. High risk for bleeding was arbitrarily defined as a platelet count of less than 40 × 10^9^/L, an activated partial thromboplastin time (aPTT) of more than 60 s, a prothrombin time of more than 2.0 international normalized ratio, a recent major bleeding, or active bleeding. Because all patients were treated according to local standards, the need for informed consent was waived for this study. Patients were also recruited from a second study, which is a randomized controlled trial initiated in 2006 (the so called ‘Citrate anticoagulation versus systemic heparinization’, CASH trial, clinicaltrials.gov number NCT00209378) [13]. Patients admitted to the ICU of the VU University Medical Center who developed AKI necessitating CVVH without a high bleeding risk, were randomised between unfractionated heparin as anticoagulant (*n* = 8) or citrate (*n* = 11). Informed consent was obtained from all study participants or their next-of-kin. We pooled the studies because of the mechanistic nature of the current paper, without focus on patient-centered outcomes.

### Treatment protocol

The indication for CVVH was based on standard clinical criteria that include AKI accompanied by hemodynamic instability, ongoing hypercatabolism, diuretic-resistant fluid overload, respiratory distress, multiorgan failure, or some combinations of these factors. CVVH was performed using a hemofiltration machine (DIAPACT, B. Braun Medical, Melsungen, Germany). Vascular access was obtained by the insertion of an 11 F double-lumen catheter (GamCath^tm^, Gambro, Hechingen, Germany) into the femoral, subclavian, or jugular vein. A 1.9 m^2^ highly permeable cellulose triacetate filter (NIPRO UF-205, Nissho Corp, Osaka, Japan, cut-off approximately 40 kDa) was used in all treatments. For lactate- or bicarbonate-based CVVH, commercially prepared buffer solutions were used (BH504 or HF32bic respectively, Dirinco, Rosmalen, the Netherlands). Patients with high serum lactate levels (>5 mmol/L) were routinely treated with bicarbonate-buffered rather than lactate buffered CVVH. For the use of citrate, a replacement solution was custom-made by Dirinco (Rosmalen, the Netherlands). Blood flow rate was set at 180 mL/min in all groups. Replacement fluid was administered at a standard rate of 2000 mL/h in the patients receiving no anticoagulation or heparin with bicarbonate or lactate-containing replacement fluids. Replacement fluid rate in the citrate group was set at 2400 mL/h and in all cases replacement fluids were infused in predilutional mode. Patients on heparin were administered prefilter a heparin bolus of 5000 IU followed by a body weight based continuous infusion targeting an aPTT between 45 and 55 s. Patients receiving citrate-based therapy had a separate intravenous infusion with calcium glubionate (Calcium Sandoz, containing 0.225 mmol/mL calcium, Novartis Consumer Health, Breda, The Netherlands). Calcium administration was adapted to concentrations of ionized calcium in the patient by a specially designed algorithm, as described before [12]. The target ionized calcium concentration in the circuit was 0.3 mmol/L, but not routinely monitored since this is almost uniformly achieved with the aforementioned settings [12]. Filters were used for 72 h at maximum and electively replaced if needed (*n* = 1 functioned well beyond *t* = 72 h).

### Study protocol

At inclusion, demographic variables were recorded such as age, gender and reason of ICU admission. Assessment of disease severity on ICU admission was done according to the Acute Physiology And Chronic Health Evaluation II (APACHE II), the Simplified Acute Physiology Score II (SAPS II) and the Sequential Organ Failure Assessment score (SOFA). We collected systemic inflammatory response syndrome (SIRS) criteria: body temperature >38 °C; a heart rate of >90 beats/min; a respiratory rate of >20 breaths/min or mechanical ventilation; and white blood cell counts of <4.0 *10^9^/L or >12.0 *10^9^/L. When SIRS (two or more criteria) and an infection were present (either clinically suspected or microbiologically confirmed), patients were classified as having sepsis. Venous blood samples were collected from the hemofiltration catheter from each patient before the initiation of CVVH and administration of heparin. Heparin was given immediately after filter connection. Thereafter, blood samples were collected after 10, 60, 180 and 720 min from the pre- and post filter pole (at *t* = 180 93 % and at *t* = 720 min 68 % of filters were patent and allowed sampling). In 40/42 (96 %) patients the first filter run was studied; in two patients a later filter run was studied because of technical reasons.

Prefilter blood was invariably drawn before addition of replacement fluids and thus reported concentrations of the markers at inlet are not diluted. Leukocytes, platelets and serum creatinine concentrations were measured before initiation of CVVH and routinely thereafter. In all patients, a zero fluid balance was achieved during the time points at which blood samples were drawn. Ultrafiltrate samples were collected from the appropriate ports. Samples were collected in standard Vacutainer tubes (Becton, Dickinson and Company, Erembodegem, Belgium) with ethylenediaminetetraacetic acid (EDTA), benzamidine and soybean trypsin inhibitor added. Samples were centrifuged at 1,300 g for 10 min at 4 °C and stored at−80 °C until assayed.

### Measurements and calculations

Concentrations of TWEAK (molecular weight (Mw) 30–35 kDa, in healthy adults 34–500 pg/mL [14]), Ang-2 (Mw 57–70 kDa, in healthy adults 0.3–2.6 ng/mL [15]), and PTX-3 (Mw 40–45 kDa, in healthy adults <2 ng/mL [16]) were measured by enzyme-linked immunosorbent assays (ELISAs). Plasma samples and ultrafiltrate were measured in separate assays with standards prepared in a 0.5 % bovine albumin serum phosphate buffered saline tween solution or fresh ultrafiltrate as appropriate. Commercially available antibody duosets were used (for TWEAK: Human TWEAK/TNFSF12, R&D Systems, UK, DY1090, for Ang-2: Human Ang-2, R&D Systems, UK, DY623, for PTX-3: Human pentraxin 3-TSG-14, R&D Systems, UK, DY1826). All measurements were done according to the protocols provided by the manufacturer. Each sample was run in duplicate and the mean concentration was calculated. Formulas used to evaluate fluxes are described in Table 1.

**Table 1:** Formulas used to evaluate fluxes

Q_i_ = Q_b_ x (1-Ht), Q_o_ = Q_i_
M_i_ = Q_i_ x Ci
M_o_ = Q_o_ x C_o_
M_uf_ = Q_uf_ x C_uf_
M_tr_ = M_i_ - M_o_
C = Ci x Q_i_/(Q_i_ + RF)
SC = 2 × C_uf_/(C + C_o_)

*Ci* Concentration in inlet plasma before addition of replacement fluid (ng/mL)

*C*_*o*_ Concentration in outlet plasma (ng/mL)

*C*_*uf*_ Concentration in ultrafiltrate (ng/mL)

*C* Concentration in inlet plasma after addition of replacement fluid (ng/mL)

*Q*_*b*_ Inlet blood flow rate (mL/min)

*Q*_*i*_ Inlet plasma flow rate (mL/min)

*Q*_*o*_ Outlet plasma flow rate (mL/min)

*Q*_*uf*_ Ultrafiltration flow rate (mL/min)

*M*_*i*_ Mass inlet rate (ng/min)

*M*_*o*_ Mass outlet rate (ng/min)

*M*_*uf*_ Mass ultrafiltration rate (ng/min)

*M*_*tr*_ Total mass removal rate (ng/min)

*RF* Replacement fluid flow rate (mL/min)

*SC* Sieving coefficient

### Statistical analysis

Due to mostly non-Gaussian distributions, data are presented as median and range. Since there were no baseline differences between the two citrate groups (citrate vs. no-anticoagulation and citrate vs. heparin), the citrate data were pooled. When appropriate, data were log-transformed to achieve normal distributions. The values for the total mass removal rate were ranked, since some values were negative and could not be log-transformed. Group differences were evaluated using *X*^2^ or Kruskal-Wallis tests, where appropriate. To evaluate differences according to anticoagulation regimens in time we used generalized estimating equations (GEE) taking repeated measurements in the same patient into account. The focus of GEE is on estimating differences between anticoagulation groups and time points, and their first order interaction, i.e. differences between anticoagulation groups over time, and associated P values are reported. Spearman correlation coefficients were used to express relations. A P <0.05 was considered statistically significant. Exact P values are given unless <0.001.

## Results

### Patient characteristics

**Table 2:** Patient characteristics

	No anticoagulation	Heparin	Citrate	
	*n* = 13	*n* = 8	*n* = 21	P
Age, yrs	70 (34–84)	57 (23–81)	64 (32–84)	0.49
Sex, male	7 (54)	6 (75)	14 (75)	0.59
Weight, kg	70 (50–100)	74 (55–135)	78 (60–110)	0.41
Sepsis	5 (39)	5 (63)	8 (38)	0.46
APACHE II at ICU admission	28 (11–42)	22 (15–37)	25 (14–41)	0.47
SAPS II at ICU admission	75 (43–112)	47 (37–77)	52 (32–86)	0.002
Creatinine, μmol/L	249 (100–410)	420 (156–626)	326 (47–622)	0.01
Urea, mmol/L	21 (6–48)	32 (12–97)	21 (6–41)	0.25
Leukocytes, ×10^9^/L	8 (1–17)	12 (7–20)	12 (1–26)	0.29
Platelets, ×10^9^/L	65 (22–173)	167 (44–352)	118 (35–332)	0.03
Prescribed CVVH dose, mL/kg/h	22 (16–32)	22 (11–32)	23 (16–31)	0.68

Median (and range) or number (percentage). *APACHE II* Acute Physiology and Chronic Health Evaluation II, *ICU* intensive care unit, *SAPS II* Simplified Acute Physiology Score II

At baseline, all patients included in this study (*n* = 42) met the criteria for SIRS and 18 of 42 (43 %) patients met the criteria for sepsis. The no anticoagulation group had higher disease severity scores than the other groups (Table 2).

### TWEAK

**Fig. 1:**
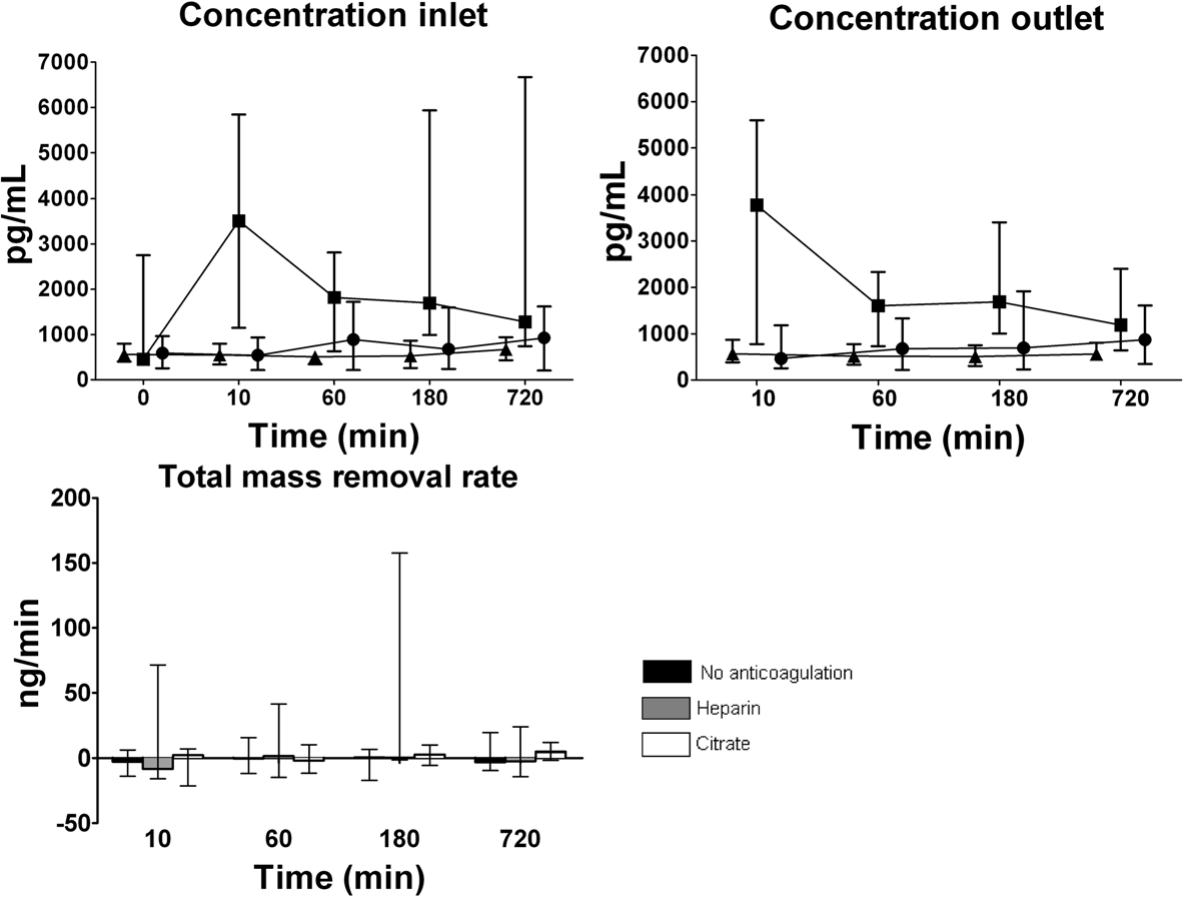
Concentration (median and interquartile range) of TWEAK during CVVH over time at inlet (before addition of replacement fluid) and outlet and the total mass removal rate. Symbols: ● no anticoagulation, ■ heparin, ▲ citrate. Concentrations at inlet and outlet were higher in the heparin group (*P* = 0.043 and *P* = 0.001, respectively) and decreased over time as compared to the other groups (*P* = 0.001 and *P* = 0.007, respectively)

Concentrations at in- and outlet were higher in the heparin group than other groups (*P* = 0.043 and *P* = 0.001, respectively), especially after 10 min of CVVH, and concentrations at in- and outlet decreased over time in the heparin group but not in the other groups (*P* = 0.001 and *P* = 0.007, respectively). TWEAK was not detectable in the ultrafiltrate and the total mass removal rate and thus absorption were low and did not differ between anticoagulation groups (Fig. 1).

### Ang-2

**Fig. 2:**
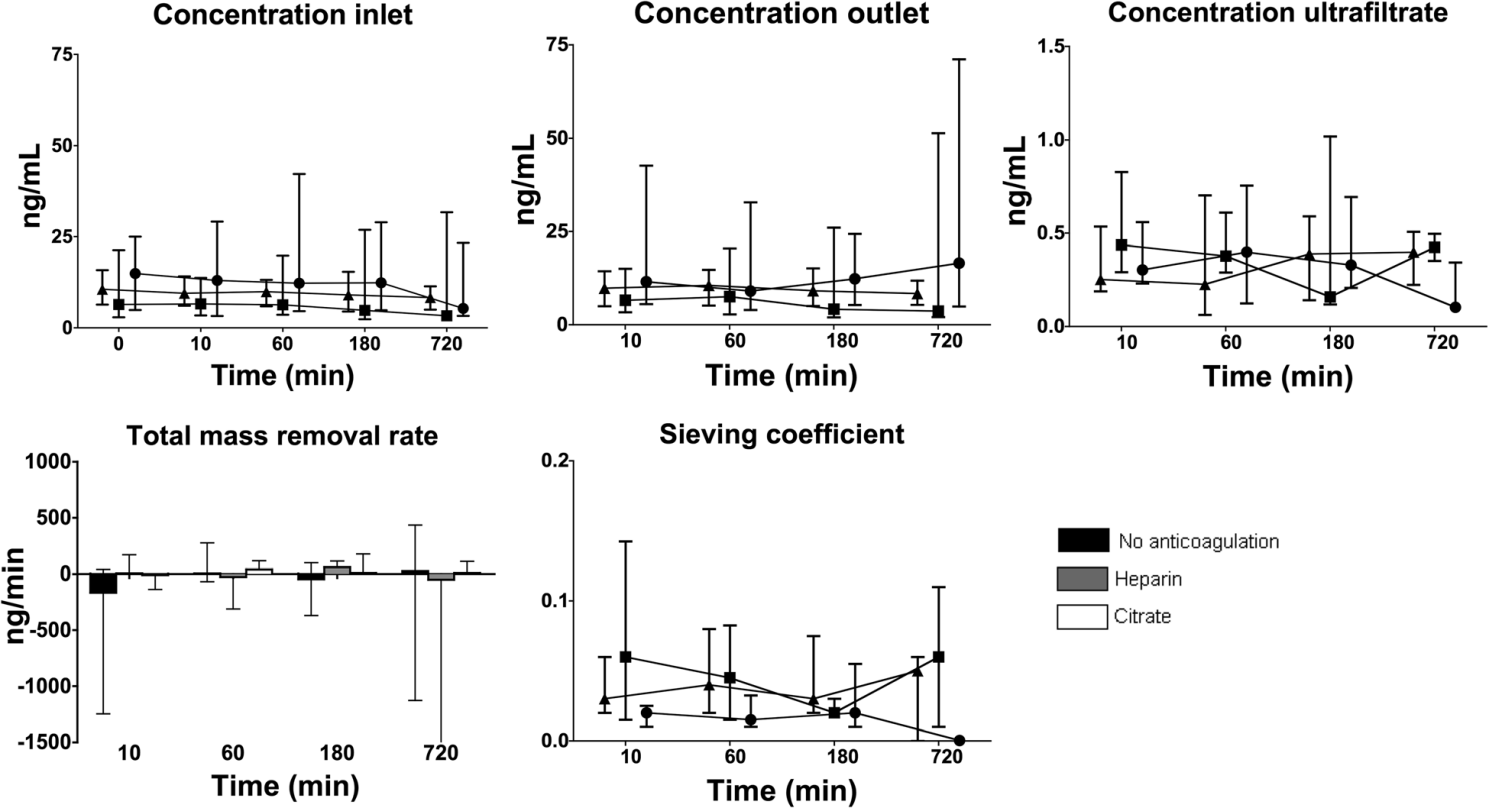
Concentration of angiopoietin-2 during CVVH over time at inlet (before addition of replacement fluid), outlet, ultrafiltrate, and total mass removal rate and sieving coefficient (median and interquartile range). Symbols: ● no anticoagulation, ■ heparin, ▲ citrate. The concentration at inlet minimally decreased over time, irrespective of anticoagulation (*P* = 0.001). The concentration in ultrafiltrate decreased over time in the no anticoagulation group, but not in the other groups (*P* < 0.001). The sieving coefficient decreased in the no anticoagulation group, but not in the others groups (*P* < 0.001 for interaction anticoagulation and time)

Concentrations at inlet minimally decreased over time in all groups (*P* = 0.001) and concentrations at outlet were similar to those at inlet, yet did not change over time in any of the groups. Ang-2 was detectable in 43 % of the total measurements in the ultrafiltrate; concentrations were low, approximately 4 % of inlet levels, and concentrations somewhat decreased over time in the no anticoagulation group, but not the other groups (*P* < 0.001). The sieving coefficient was low with medians ranging from 0.01 to 0.1 and decreased over time in the no anticoagulation group, but not the other groups (*P* < 0.001). Also, the total mass removal rate was zero, since concentrations of Ang-2 in the ultrafiltrate were negligible compared to in- and outlet concentrations, and did not differ between groups over time. Hence, there was no absorption either (Fig. 2).

### PTX3

**Fig. 3:**
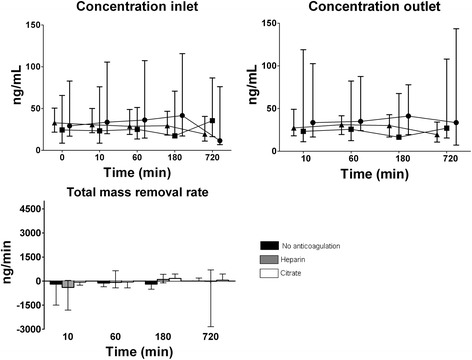
Concentration (median and interquartile range) of pentraxin-3 (PTX-3) during CVVH over time at inlet (before addition of replacement fluid), outlet and the total mass removal rate. Symbols: ● no anticoagulation, ■ heparin, ▲ citrate. Concentrations at inlet decreased over time in the no anticoagulation and citrate group, but not in the heparin group (*P* = 0.009 for interaction anticoagulation and time)

Concentrations at inlet minimally decreased over time in the no anticoagulation and citrate group, but not in the heparin group (*P* = 0.009 for interaction anticoagulation and time). Concentrations at outlet were similar and did not change over time in any of the anticoagulation groups. PTX3 was not detectable in the ultrafiltrate and the total mass removal rate (and thus absorption) was low and did not differ between anticoagulation groups (Fig. 3).

### Correlations

There was no correlation between TWEAK, Ang-2 or PTX3 and creatinine levels at initiation of CVVH. Plasma levels of TWEAK, Ang-2 and PTX3 at inlet at the start of CVVH correlated with those at the end of the CVVH run (r_s_ = 0.68, r_s_ = 0.85, r_s_ = 0.73, respectively, all *P* < 0.001), suggesting good reproducibility of measurements.

### Sepsis and mortality

Concentrations of TWEAK were higher in patients with sepsis (606 (236–28549) pg/mL) than in patients without sepsis (493 (121–2181, *P* = 0.011). 16 of the 42 (38 %) patients died in the ICU. Plasma levels of Ang-2 and PTX3 at inlet were higher in non-survivors at initiation of CVVH: 16 (1.2–230) vs 9.6 (0.6–34) ng/mL, *P* = 0.019 and 34 (7.4–235) vs 27 (6.9–187) ng/mL, *P* = 0.015, respectively.

## Discussion

The present study shows that plasma levels of putative novel AKI mediators in critically ill patients are not affected by CVVH; there is no clearance, absorption nor production across the system. Although inlet (patients plasma) concentrations of Ang-2 and PTX3 are slightly declining in the study interval, there is no or hardly any net removal by CVVH. However, heparin anticoagulation increases TWEAK levels in the patient whereas citrate does not, favouring the latter as anticoagulation during CVVH. Indeed, the result may help to explain less rapid AKI recovery when nadroparin is used for anticoagulation during CVVH, as compared to citrate [17].

The reason that heparin is associated with transiently increased TWEAK in the patients, especially early after administration, is conjectural but may relate to proinflammatory actions of high concentrations of heparin [18]. Platelet activation by heparin may also play a role since TWEAK is also stored in platelets [19]. High concentrations of TWEAK are undesirable, especially in an inflammatory microenvironment, and neutralizing anti-TWEAK antibodies, as a tissue protection strategy in kidney disease, are currently being explored [20]. Even though the concentration of TWEAK decreased over time in the heparin group, there was no net removal of TWEAK by CVVH, since in- and outlet concentration were similar. Also, this suggests the increase of TWEAK by heparin early in CVVH occurs in the patients rather than in the filter or extracorporeal circuit.

We cannot explain, except by effects of spherical configuration and electrical load of the molecules why Ang-2, a relatively large molecule, was found in the ultrafiltrate, albeit at low concentrations, and TWEAK and PTX3 with molecular weight at or below the membrane cutoff were not. Although the concentration of Ang-2 decreased slightly at inlet but not outlet, there was no evidence of production of Ang-2 in the filter. Also, there was no significant production of PTX3 in the filter, thus concentrations at inlet did not increase during the CVVH run, in contrast to a previous study in which PTX3 increased after a single session of hemodialysis [21]. Therefore, concentrations of Ang-2 and PTX3 were not affected by CVVH, irrespectively of the anticoagulation method.

The mediators studied bare some relation with sepsis and mortality, supporting their biological importance in the critically ill. However, we could not establish a relation with creatinine to suggest a mediator role of circulating factors in the development of AKI in our patients, but confounding factors may be involved, such as time factors, patient selection, muscle mass and others. In any case the levels of these mediators were supranormal in our patients cohort.

The limitations of the present study include the relatively small size of groups and the absence in part of randomisation, explaining some baseline differences among the groups. Patients in the no anticoagulation group did not receive anticoagulation because of a bleeding tendency and, indeed, were more severely ill at baseline and lower platelet counts. We cannot exclude earlier start of CVVH in the no anticoagulation group than in the other groups, since initial creatinine was lower, but this does not invalidate our conclusions of our pathophysiologic study. Furthermore, we have evaluated the course of the study variables for a period up to 12 h only, and do not exclude changes beyond that time interval.

## Conclusions

In conclusion, plasma levels of three putative novel AKI mediators (TWEAK, Ang-2, PTX3) in critically ill patients are not affected by CVVH, since clearance and absorption by the filter were low. Nevertheless, heparin treatment was associated with a rise in TWEAK concentrations whereas citrate was not, suggesting that citrate is more suitable for anticoagulation in CVVH for AKI.

## Abbreviations

AKI: Acute kidney injury
Ang-2: Angiopoietin-2
APACHE II: Acute physiology and chronic health evaluation II
aPTT: Activated partial thromboplastin time
CVVH: Continuous venovenous hemofiltration
EDTA: Ethylenediaminetetraacetic acid
ELISA: Enzyme-linked immunosorbent assays
GEE: Generalized estimating equations
ICU: Intensive care unit
PTX3: Pentraxin-3
SAPS II: Simplified acute physiology score II
SIRS: Systemic inflammatory response syndrome
SOFA: Sequential organ failure assessment score
TNF: Tumor necrosis factor
TWEAK: TNF-associated weak inducer of apoptosis
